# ENRICHING OUR DISCOVERY BY TRAINING FOR ENGAGEMENT: METHODS FOR PATIENT/PERSON ENGAGEMENT IN RESEARCH

**DOI:** 10.1093/geroni/igac059.1276

**Published:** 2022-12-20

**Authors:** Rachel Lessem, Martina Roes, Katherine Abbott

## Abstract

There has been a recent movement towards greater involvement of the patient/person voice in aging research. However, many researchers embark on engaged research without adequate training or knowledge and without the skills or experience to train those they hope to engage. We define engaged research as research topics, designs, methods, analyses and/or dissemination strategies that have been informed through collaboration with or input from those who have a direct stake in research. This symposium explores education as a method of engaged research and is premised on the notion that engaged research enriches our discovery and ultimately leads to better outcomes in interventions as well as policy. There is an identified need for training of researchers as well as stakeholders in order to do successful engaged research. This symposium details educational methods for training those who utilize engaged research: student, researcher, and patient/person stakeholder, culminating in an examination of supporting patients in being trainers themselves. The first presentation investigates an overarching educational strategy to change the research paradigm. The second presentation explores a unique method for training researchers to engage older adults as advisory board members. The third presentation details a strategy for training a specific group of older adults to be active in engaged research. The symposium concludes by highlighting a method by which older adults engage in a research intervention as trainers. Attendees will be able to identify and evaluate the outcomes of the different educational methods and translate these methods to new settings.

